# Planar-Twisted Molecular Engineering for Modulating the Fluorescence Brightness of NIR-II Fluorophores with a Donor–Acceptor–Donor Skeleton

**DOI:** 10.3390/ijms252212365

**Published:** 2024-11-18

**Authors:** Shengjiao Ji, Yuying Du, Jiancai Leng, Yujin Zhang, Wei Hu

**Affiliations:** International School for Optoelectronic Engineering, School of Chemistry and Chemical Engineering, Qilu University of Technology (Shandong Academy of Sciences), Jinan 250353, China; ji1051434804@163.com (S.J.); 18753685661@163.com (Y.D.); jiancaileng@qlu.edu.cn (J.L.)

**Keywords:** fluorescence brightness, NIR-II fluorophore, planar-twisted strategy

## Abstract

Organic molecular fluorophores have been extensively utilized for biological imaging in the visible and the first near-infrared windows. However, their applications in the second near-infrared (NIR-II) window remain constrained, primarily due to the insufficient fluorescence brightness. Herein, we employ a theoretical protocol combining the thermal vibration correlation function with the time-dependent density functional theory method to investigate the mechanism of the planar-twisted strategy for developing fluorophores with balanced NIR-II emission and fluorescence brightness. Based on a planar donor–acceptor–donor molecular skeleton, various ortho-positioned alkyl side chains with steric hindrances are tactfully incorporated into the backbone to construct a series of twisted fluorophores. Photophysical characterizations of the studied fluorophores demonstrate that the emission spectra located in the NIR-II region exhibited a hypsochromic shift with the structural distortion. Notably, conformational twisting significantly accelerated the radiative decay rate while simultaneously suppressing the nonradiative decay rate, resulting in an improved fluorescence quantum efficiency (FQE). This enhancement can be mainly attributed to both the enlarged adiabatic excitation energy and reduced nonadiabatic electronic coupling between the first excited state and the ground state. Compared with the planar fluorophore, the twisted structures possessed a more than fivefold increase in FQE. In particular, the optimal twisted fluorophore BBTD-4 demonstrated a desirable fluorescence brightness (16.59 M^−1^ cm^−1^) on the premise of typical NIR-II emission (980 nm), making it a promising candidate for NIR-II fluorescence imaging in biomedical applications. The findings in this study elucidate the available experimental observations on the analogues, highlighting a feasible approach to modulating the photophysical performances of NIR-II chromophores for developing more highly efficient fluorophores toward optical imaging applications.

## 1. Introduction

Near-infrared fluorescence imaging is an emerging technology gaining significant attention in biomedicine due to its specificity, cost-effectiveness and noninvasive and real-time capabilities [1,2]. This imaging modality can be categorized into NIR-I (700–900 nm) and NIR-II (900–1700 nm) protocols based on the emission wavelength [3,4]. While NIR-I fluorescence imaging outperforms visible light imaging, its limited tissue penetration depth poses challenges for broader applications. In contrast, NIR-II fluorescence imaging excels with superior temporal and spatial resolutions, a high signal-to-noise ratio and deep tissue penetration, benefiting from reduced absorption, scattering and low autofluorescence [5,6]. During the initial application of NIR-II bioimaging for guiding surgeries in liver cancer patients, intraoperative assessments have demonstrated a remarkable improvement in sensitivity and lesion identification compared with the imaging performed in the visible and NIR-I regions [7,8,9,10]. For facilitating efficient, accurate and noninvasive NIR-II fluorescence imaging, a variety of NIR-II chromophores have been specifically developed for imaging-mediated phototherapy [11,12,13].

To date, materials including carbon nanotubes, semiconductor quantum dots, rare earth-doped nanoparticles and nanoclusters have been explored for NIR-II fluorescence imaging [14,15]. However, concerning the potential long-term toxicity of the inorganic agents in clinical applications, there is growing interest in organic fluorophores among researchers due to their high biocompatibility, low cytotoxicity and rapid excretion [16,17,18]. Currently, one of the focuses in bioimaging research is the development of NIR-II molecular dyes with a donor–acceptor–donor (D–A–D) motif [19,20]. This molecular skeleton effectively narrows the energy gap between the highest occupied molecular orbital (HOMO) and the lowest unoccupied molecular orbital (LUMO) of the fluorophore, leading to a bathochromic shift in the fluorescent band and potentially extending the emission into the NIR-II region or beyond.

In addition to a long emission wavelength, considerable fluorescence brightness is also essential for an efficient NIR-II molecular fluorophore. Unfortunately, most reported NIR-II fluorophores suffer from low fluorescence quantum efficiency (FQE) or molar absorption coefficients in comparison with visible and NIR-I chromophores, resulting in inferior fluorescence brightness. This shortcoming greatly hinders the effectiveness of NIR-II biosensing and bioimaging applications, presenting significant obstacles to their development and clinical implementation. Thus, up to now, developing NIR-II fluorescent probes with high brightness is still challenging.

Recent studies have demonstrated the effect of spacers between donor and acceptor moieties on the luminescence performance of NIR-II fluorophores. For instance, based on IR-E1 with a benzo [1,2-c:4,5-c′]bis([1,2,5]thiadiazole) (BBTD) acceptor, the NIR-II fluorophore IR-BGP6 was developed by replacing the π-bridge with a tert(ethylene glycol)-substituted thiophene, which exhibited a notably higher FQE (0.15%) than IR-E1 (0.07%) [21,22]. Additionally, by utilizing BBTD as the acceptor and fluorene derivative as the donor, 3,4-ethoxylene dioxythiophene or 3-(2-(2-(2-methoxyethoxy)ethoxy)ethoxy)-substituted thiophene were incorporated into the skeleton to develop the NIR-II dyes IR-FTX and IR-FEXP [23]. Subsequently, Ma et al. synthesized another NIR-II fluorophore, IR-FP8P, which features a novel spacer comprising dioctyl chains in 3,4-propylenedioxy thiophene [24] and exhibits an emission peak at 1040 nm with a remarkable 3.7 fold increase in brightness over IR-FTAP. Alkyl thiophene has been demonstrated to induce backbone distortion in the conjugated molecule, which would enhance the fluorescence quantum yield of D–A–D-type NIR-II fluorophores. However, the highly twisted structure inevitably destroys the molecular conjugation, thus effectuating an inferior absorption coefficient and fluorescence brightness. Therefore, rational modulation of the molecular structures of NIR-II fluorophores to achieve an optimal balance between the absorption intensity and luminescent efficiency is valuable for further improving the fluorescence brightness.

Herein, we investigate the inner mechanism of the planar-twisted molecular engineering, aiming at enhancing the brightness of NIR-II fluorophores from a theoretical perspective. On the basis of the D–A–D molecular framework, alkyl side chains are incorporated into the thiophene spacer to design BBTD-*n* fluorophores (*n* = 1, 2, 3, 4). These molecules display a variety of conformations ranging from planar to highly twisted geometries compared with the experimental analogues [23,24]. The molecular distortion effect on the absorption properties and fluorescent performance is investigated. Our findings indicate that both the FQE and fluorescence brightness are monotonically enlarged with molecular twisting. These improvements can be mainly attributed to the simultaneously elevated adiabatic excitation energy and reduced nonadiabatic electronic coupling, which are relevant to the restricted C-H in-plan bending within the π-bridge unit. In particular, BBTD-4, with the largest distortion, achieved the highest fluorescence efficiency and brightness with efficient NIR-II emission. The present research focuses on the effect of conformational twisting on the luminescent performance of NIR-II chemosensors, revealing the structure–property relationships and providing a theoretical basis for developing advanced organic NIR-II luminescent dyes with superior brightness in biomedical fields.

## 2. Results and Discussion

### 2.1. Design Strategy and Molecular Conformation

**Figure 1:**
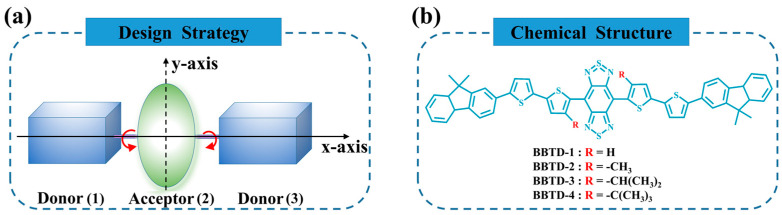
(**a**) Design strategy and (**b**) chemical structures of BBTD-*n* (*n* = 1, 2, 3, 4).

Following the design strategy shown in Figure 1a, a series of fluorophores with varying degrees of distortion were constructed by utilizing the molecular skeleton provided by the experimental group [23,24]. In the conjugated D–A–D molecules, fluorene derivatives were selected as donor units, and 2,2′-bithiophene acted as both the π conjugation unit and the electronic bridge for facilitating the intramolecular charge transfer. In addition, an extremely electron-deficient moiety, benzo [1,2-c:4,5-c’]bis ([1,2,5]thiadiazole) (BBTD), was adopted as the acceptor to construct near-infrared emission compounds, which are named BBTD-*n* (*n* = 1, 2, 3, 4). Notably, different alkyl side chains were introduced into the thiophene unit to construct twisted molecular structures. To be specific, the hydrogen atoms in BBTD-1 were replaced by the methyl, isopropyl and tert-butyl groups in BBTD-2, BBTD-3 and BBTD-4, respectively. In the previous works, bulky spacers such as 3,4-ethylenedioxy thiophene (EDOT) and dioctyl chain-substituted 3,4-propylenedioxy thiophene (PDOT) were incorporated into the D–A–D architecture to distort the conjugated backbone for tuning the fluorescence performance. Herein, alkyls were adopted in the design strategy due to their simplified and cost-effective synthesis, as well as the facile modification on the length or branch structures, to introduce diversified steric hindrances.

**Figure 2:**
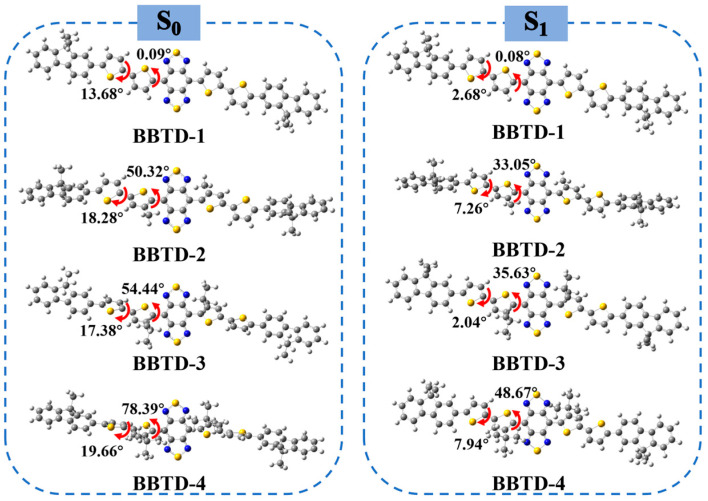
Optimal molecular geometries in the ground state (S_0_) and in the first excited state (S_1_).

Figure 2 illustrates the optimized geometries of the molecular fluorophores in the S_0_ and S_1_ states. In the ground states, the dihedral angle between the BBTD and thiophene increased gradually with enlarged steric hindrance of the side chain, being 0.09° for BBTD-1, 50.32° for BBTD-2, 54.44° for BBTD-3 and 78.39° for BBTD-4. However, the dihedral angles between the thiophene and fluorene units were similar, ranging from 13.68° to 19.66° in the BBTD-*n* fluorophores. By summing up these two dihedral angles, it was observed that the torsion between the π-bridge unit and the acceptor or donor followed the order of BBTD-4 > BBTD-3 > BBTD-2 > BBTD-1. Relatively, all dihedral angles decreased in the S_1_ geometries, indicating a geometric reorganization in the excited state which facilitated the planarization and electron delocalization of the π-conjugated backbone. It is worth noting that BBTD-4 exhibited a significantly larger reduction in its torsion angle (29.72°) between the acceptor and thiophene spacer than BBTD-1 (0.01°), BBTD-2 (17.27°) and BBTD-3 (18.81°). This suggests greater conformational variation during the electronic transfer process, leading to more noticeable changes in the photophysical characteristics.

### 2.2. Photoabsorption and Photoemission Properties

**Figure 3:**
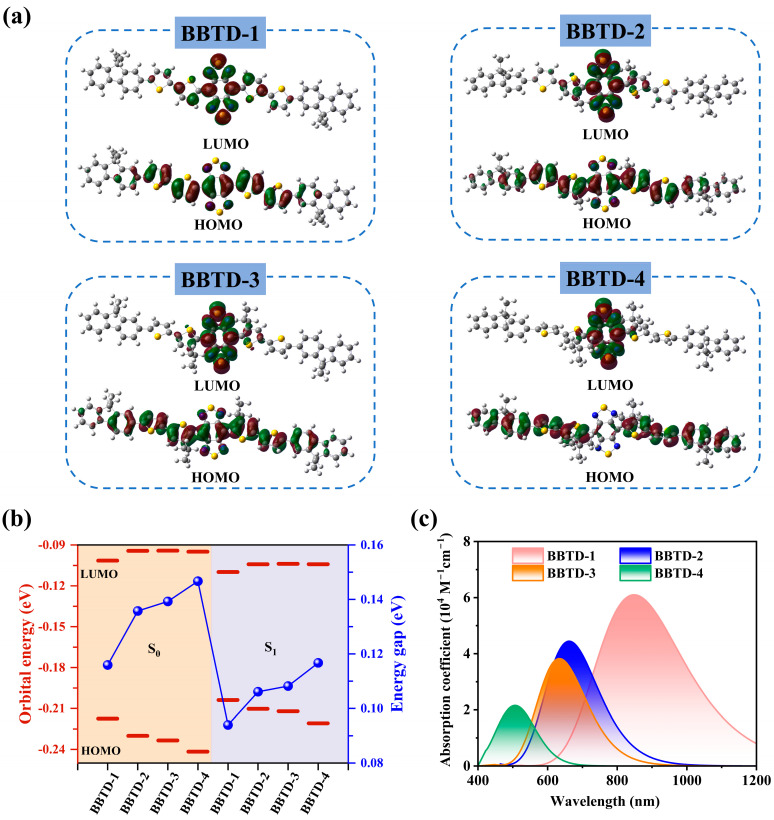
(**a**) Distributions of HOMO and LUMO, (**b**) orbital energy levels with energy gaps and (**c**) absorption spectra for BBTD-*n* (*n* = 1, 2, 3, 4).

To shed light on the effect of molecular distortion on the photophysical performance of the investigated fluorophores, the nature of the S_1_ state was further analyzed. Firstly, the frontier molecular orbitals of the compounds are presented in Figure 3a. The electronic distributions of the LUMOs for the BBTD-*n* compounds were mainly localized on the BBTD unit, while the HOMOs were delocalized across the entire conjugated backbone, suggesting a strong D–A effect and intramolecular charge transfer from the electron-donating thiophene units to the electron-accepting BBTD core. It is worth noting that in the HOMO of BBTD-4, there was almost no electronic distribution on the BBTD moiety due to the highly twisted molecular backbone. Compared with the nearly co-planar BBTD-1, the LUMO energy levels of the twisted fluorophores were elevated, and the HOMO energy levels were successively lowered to an even greater extent. As a result, the HOMO–LUMO gaps of the twisted fluorophores were relatively larger, especially in the most distorted conformational BBTD-4. Upon excitation, the HOMO levels of the studied molecules increased significantly, while the LUMO levels experienced slight decreases, leading to a narrower HOMO–LUMO gap. The planar BBTD-1 architecture further lowered the LUMO level and simultaneously elevated the HOMO level even more. This pronounced increase in the HOMO level largely reduced the energy gap, which is crucial for achieving bathochromic shifts in optical processes.

Motivated by the distinct geometric and electronic structures of the BBTD-*n* fluorophores, the photophysical properties of BBTD-1–4 with planar to twisted skeletons were investigated, and the crucial parameters were collected in Table 1. For all of the studied compounds, the absorption bands in an aqueous solution were found to cover the traditional visible light to the NIR-I region, displaying an evident hypsochromic shift with increasing distortion of the molecular skeleton (Figure 3c). Due to the steric hindrance of the ortho-positioned tert-butyl substituent, BBTD-4 exhibited a maximum absorption wavelength ($\lambda_{\mathrm{abs}}^{\max}$) at 504 nm, while that of BBTD-1 with a higher π conjugation extent was significantly red-shifted to 848 nm. Correspondingly, the $\lambda_{\mathrm{abs}}^{\max}$ values of BBTD-2 and BBTD-3 were located at 662 and 634 nm, respectively. In addition, the maximum molar extinction coefficients were determined to be 6.10 × 10^4^, 4.44 × 10^4^, 3.82 × 10^4^ and 2.15 × 10^4^ M^−1^ cm^−1^ for BBTD-1–4, respectively. This decreasing value in the absorption intensity can be attributed to the increased torsion of the conjugated backbones and the anabatic localization of the LUMO. It is evident that the twisted molecular skeleton was less favorable than the planar conjugated one for applications involving absorptive response. These findings align with the experimental measurements of the BBTD-*n* analogues [23,24] and further confirm the positive effect of coplanar conformation in enhancing the molecular light harvesting ability.

**Figure 4:**
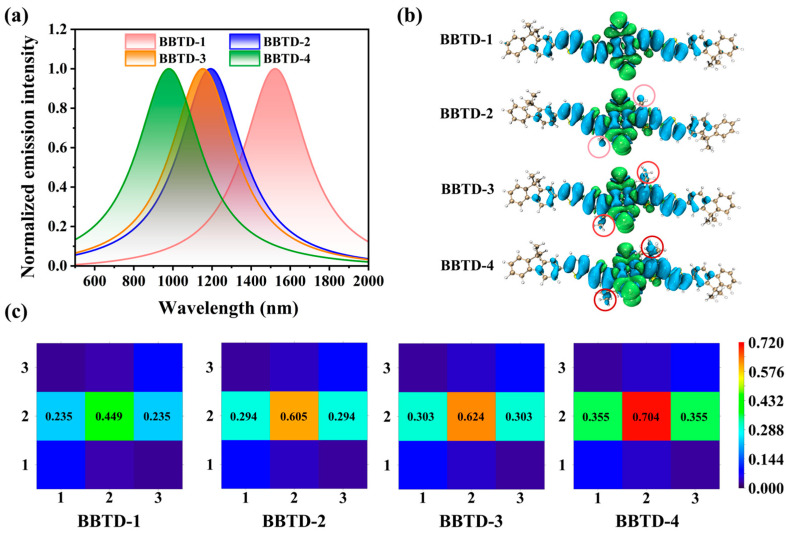
(**a**) Normalized emission spectra, (**b**) intramolecular charge transfer from S_1_ to S_0_ (cyan represents holes, and green represents electrons) and (**c**) transition density matrices for BBTD-*n* (*n* = 1, 2, 3, 4).

As expected, the normalized photoemission spectra of the investigated fluorophores were prominently extended to the NIR-II window, highlighting their potential for in vivo NIR-II fluorescence imaging applications. The maximum fluorescent peaks for BBTD-1–4 were located at 1522, 1192, 1153 and 980 nm, respectively (Figure 4a). This bathochromic shift correlated with the planarization of the molecular backbone, aligning with the similar trends noted in the absorption spectra. Furthermore, the transition nature associated with the emission processes for the compounds was examined. As depicted in Figure 4b, the overall transition characteristics of the studied fluorophores were notable for their resemblance. In general, during the transition from the S_1_ to S_0_, the electrons which were primarily distributed on the donor moieties were mainly transferred to the acceptor unit, demonstrating a typical intramolecular charge transfer feature. This electronic transition process could be intuitively characterized by a transfer from holes to electrons, indicating the intricate electronic interactions within the fluorophores. Notably, in the case of BBTD-2–4, additional holes were found to be distributed on the alkyl side chain substituents. This observation suggests variations in the transition mechanisms among different fluorophores, highlighting the subtle differences in their photophysical properties.

By dividing the molecules into three fragments (as shown in Figure 1a)—a donor moiety, labeled fragment 1, an acceptor moiety, labeled fragment 2, and another donor moiety, labeled fragment 3—the transition density matrix (TDM) of the S_1_-to-S_0_ transition is quantitatively evaluated by $T(r;r’)=\sum_{i}\sum_{a}w_{i}^{a}\varphi_{i}(r)\varphi_{a}(r’)$. Here, *a* and *i* represent the indexes of the unoccupied and occupied orbitals, respectively, and φ(r) is the configuration function with a coefficient of *w*. The diagonal element of the hot maps in Figure 4c reflects the distributions of both the electrons and holes, while in the non-diagonal element, the horizontal and longitudinal coordinates correspond to the hole distribution and electron distribution, respectively. For all BBTD-*n* compounds, the emission processes were primarily contributed by transitions localized on segment 2, with slight involvement in the electron transfer from fragments 1 and 3 to fragment 2. Relative to the planar BBTD-1, the TDM element T(2;2) for BBTD-4 reached an impressive value of 0.704. In contrast, BBTD-2 and BBTD-3 exhibited T(2;2) values of approximately 0.6, indicating a relatively limited overlap between electrons and holes in the acceptor moiety. This discrepancy can be rationalized by the highly localized LUMO distribution resulting from the distorted architecture of BBTD-4. These findings certified the availability of the planar-twisted tactic via delicate regulation of the modified group in precisely tuning the fluorescent behaviors and photophysical characteristics of NIR-II fluorophores.

### 2.3. Fluorescence Quantum Efficiency

**Figure 5:**
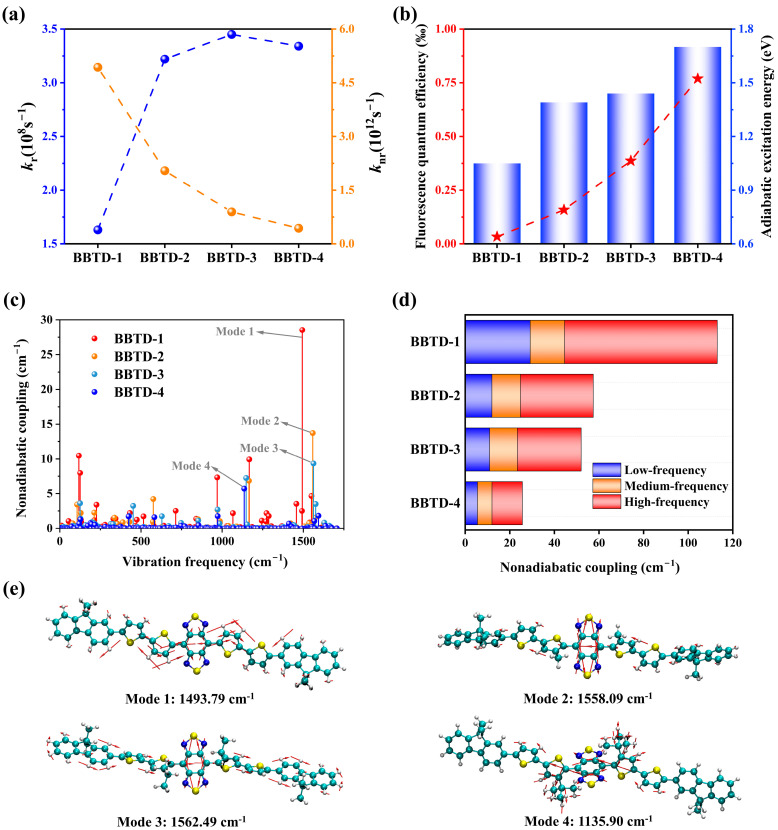
(**a**) Radiative decay rate constants (blue balls) and nonradiative decay rate constants (orange balls), (**b**) fluorescence quantum efficiency (red star) and adiabatic excitation energy (blue column) and (**c**) nonadiabatic coupling versus mode frequency with (**d**) the contributions from the low-frequency, medium-frequency and high-frequency normal modes for BBTD-*n* (*n* = 1, 2, 3, 4). Representative vibration modes labeled in (**c**) are shown in (**e**).

Luminescent efficiency is a fundamental indicator for evaluating the feasibility of a dye as an efficient NIR-II fluorophore. Thus, the radiative and nonradiative decay rate constants were calculated as plotted in Figure 5a. For the BBTD-*n* compounds, the radiative rate constants of the S_1_-to-S_0_ transition fell within the range of 1.5 × 10^8^–3.5 × 10^8^ s^−1^. According to Equations (1) and (4), the radiative rate constant was determined by the transition electric dipole moments *μ_i_* and the vertical transition energy Δ*E*_vert_. Despite the reduced *μ_i_* of 18.70, 15.55, 14.88 and 12.40 Debye for BBTD-1–4, respectively, there was a noticeable numerical enhancement of *k*_r_ as the molecular distortion increased from BBTD-1 to BBTD-2, BBTD-3 and BBTD-4, which can mainly be attributed to Δ*E*_vert_ enlarging from 0.81 to 1.04, 1.08 and 1.27 eV, respectively. Importantly, the nonradiative rate constant was largely reduced by one order of magnitude from 4.93 × 10^12^ for BBTD-1 and 2.04 × 10^12^ for BBTD-2 to 8.93 × 10^11^ for BBTD-3 and 4.34 × 10^11^ for BBTD-4 s^−1^, showing high sensitivity to the conformational twisting. Consequently, a more than fivefold enhancement in FQE was achieved in the twisted molecules, as illustrated in Figure 5b. Based on the above results, it can be concluded that the increased FQE of BBTD-*n* fluorophores is predominantly attributed to the significant reduction in *k*_nr_ for the transition from S_1_ to S_0_. This is consistent with previous research on the luminescence efficiency of organic molecules used in optical devices [25,26]. Furthermore, the observed trend of the enhanced FQE in correlation with the distortion aligned with the experimental findings on BBTD-*n* analogues [23,24].

The nonradiative rate is essentially relevant to the adiabatic excitation energy (Δ*E*_ad_), which corresponds to the energy difference between the equilibrium configurations of S_0_ and S_1_, and the nonadiabatic coupling, whose coupling matrix element is defined as $\langle \Psi_{f}|\frac{\partial }{\partial Q_{fl}}|\Psi_{i}\rangle \approx -\frac{\langle \Psi_{f}^{0}|\partial \hat{V}/\partial Q_{fl}|\Psi_{i}^{0}\rangle }{E(\Psi_{f}^{0})-E(\Psi_{i}^{0})}$ based on the first-order perturbation theory [27]. As demonstrated in Figure 5b, the Δ*E*_ad_ values for BBTD-1, BBTD-2, BBTD-3 and BBTD-4 were 1.05, 1.39, 1.44 and 1.70 eV, respectively. The monotonically increased Δ*E*_ad_ can be attributed to the rising energy difference between the HOMO and LUMO, which is favorable for reducing the driving force of internal conversion from S_1_ to S_0_, thus leading to a decrease in *k*_nr_ and an improvement in luminescence efficiency. When compared with the π-conjugated planar BBTD-1, the highly twisted BBTD-4 featuring the largest Δ*E*_ad_ is expected to exhibit a more favorable FQE according to the approximated relationship between *k*_nr_ and Δ*E*_ad_ of $\ln(k_{nr})\propto -{\left(\Delta E_{\mathrm{ad}}-\lambda \right)}^{2}/4\lambda k_{B}T$, based on the linearly displaced model and short-time approximation [28,29,30].

Upon the analysis of the nonadiabatic electronic coupling between S_1_ and S_0_ (Figure 5c,d), it was observed that (1) the primary contributions to the total nonadiabatic coupling mainly arose from the high-frequency modes (>900 cm^−1^) for all fluorophores; (2) as the molecular distortion increased, the nonadiabatic coupling was significantly reduced due to the numerical decline in the three regions, including at a low frequency (0–300 cm^−1^), medium frequency (300–900 cm^−1^) and high frequency; and (3) strikingly, the normal mode which contributed the most to the nonadiabatic coupling of BBTD-1, BBTD-2 and BBTD-3 exhibited a frequency of about 1500 cm^−1^, whereas that of BBTD-4 was much smaller. Careful inspection of these normal modes (Figure 5e) revealed that in BBTD-1, the C-H bending vibration on the thiophene adjacent to the BBTD moiety was the primary source of nonadiabatic coupling with a contribution of 28.53 cm^−1^, serving as a dominant pathway for the nonradiative transition and the dissipation of excited state energy. Conversely, the twisted skeletons of BBTD-2, BBTD-3 and BBTD-4, resulting from the attachment of alkyl side chains, effectively blocked this nonradiative decay channel. Notably, the bending motion of the C-H bond in the tert-butyl group played a dominant role in the nonadiabatic coupling of BBTD-4, with a much smaller contribution of 5.73 cm^−1^. Therefore, the improved FQE of this series of fluorophores can be partly attributed to effective inhibition of the intense vibrations associated with the planar configuration through considerable distortion, reducing the nonadiabatic electronic coupling in the S_1_-to-S_0_ transition process.

### 2.4. Fluorescence Brightness

**Figure 6:**
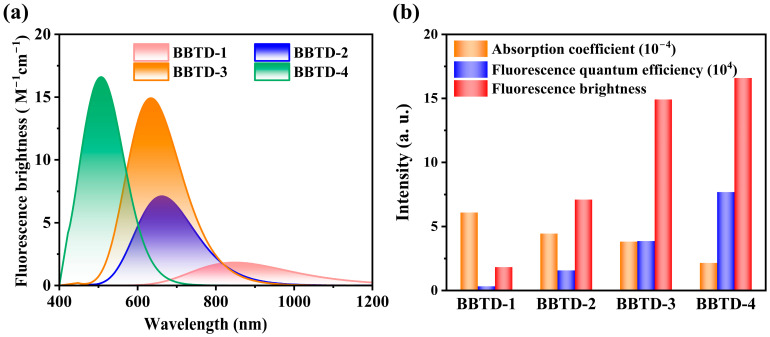
(**a**) Fluorescence brightness spectra and (**b**) comparisons on brightness maxima for BBTD-*n* (*n* = 1, 2, 3, 4).

Given that the molecular distortion was positively correlated with the absorption intensity but negatively correlated with the FQE, the integrated fluorescence brightness spectra of the investigated planar-twisted fluorophores were subsequently investigated. As displayed in Figure 6a, the major morphology of the brightness spectra closely resembled the absorption spectral pattern, particularly at the peak positions, which exhibited a continuous red-shift as the twisting of the molecular backbone increased. In light of the tunable excitation wavelength in practical fluorescence applications, the maximum brightness was selected to evaluate the NIR-II imaging capabilities of the BBTD-*n* compounds. As illustrated in Figure 6b, the brightness maxima of BBTD-1–4 were determined to be 1.83, 7.11, 14.91 and 16.59 M^−1^ cm^−1^, respectively. The distorted backbone of the BBTD-*n* fluorophores achieved significant enhancement for the brightness, highlighting the influence of structural twisting modification on the photophysical properties. More importantly, while the absorption coefficient decreased due to the molecular twisting, the increased FQE dominated the enhancement in fluorescence brightness (Figure 6b). These findings suggest that rational distortion of the structure plays a crucial role in achieving excellent luminescent characteristics, which is qualitatively consistent with the experimental observations on BBTD-*n* analogues [23,24]. With the long-wavelength NIR-II emission and high brightness exceeding 15 M^−1^ cm^−1^, BBTD-4 is prospected to be an outstanding fluorophore with promising potential for high-performance NIR-II fluorescence imaging applications.

## 3. Materials and Methods

### 3.1. Absorption and Emission Probability

The transition probabilities of photon absorption and photon emission can be represented by the dimensionless oscillator strength (*δ*) as follows:

(1) $$\delta =\frac{2\omega_{f}}{3}{\sum_{\alpha }\left|\langle 0|\mu_{\alpha }|f\rangle \right|}^{2}.$$

where |0〉 denotes the ground state, ω*_f_* is the excitation energy of the excited state |*f*〉 and *μ_α_* is the Cartesian component of the electronic dipole moment operator. Note that the summation in the equation is performed over the molecular *x*, *y* and *z* axes.

### 3.2. Fluorescence Brightness and Fluorescence Quantum Efficiency

At a fundamental level, fluorescence brightness refers to the number of photons emitted by a chromophore per unit of time upon irradiation. It is calculated based on the molar extinction coefficient (*ε*, in M^−1^ cm^−1^) and FQE of an emitter as follows:

Brightness = ε × FQE(2)

This is typically expressed in M^−1^ cm^−1^.

Herein, FQE is determined by the competition between the radiative decay rate constant (*k*_r_) and the nonradiative decay rate constant (*k*_nr_), as shown below:

(3) $$\mathrm{FQE}=\frac{k_{r}}{k_{r}+k_{\mathrm{nr}}}.$$

In the above formula, *k*_r_ can be obtained using the Einstein spontaneous emission relationship:

(4) $$k_{r}=\frac{\delta \cdot \Delta E_{\mathrm{vert}}^{2}}{1.499},$$

where Δ*E*_vert_ is the vertical transition energy in the wavenumber unit.

Additionally, according to the Condon approximation and Fermi golden rule, *k*_nr_ is written as

(5) $$k_{\mathrm{nr}}=\frac{2\pi }{\hbar }\sum_{u,v}P_{iv}|{\hat{H}}_{fu,i\nu }{|}^{2}\delta (E_{iv}-E_{fu}).$$

Here, *P_iv_* represents the Boltzmann distribution function of the initial state, and *E_iv_* (*E_fu_*) is the electronic and vibration energy of the initial (final) state. The term ${\hat{H}}_{fu,iv}$ denotes the interaction between two different states, following

(6) $${\hat{H}}_{fu,iv}\Psi_{iv}={\hat{H}}^{BO}\Phi_{i}(r,Q)\Theta_{iv}(Q)+{\hat{H}}^{SO}\Phi_{i}(r,Q)\Theta_{iv}(Q).$$

In Equation (6), *r* and *Q* represent the normal coordinates of the electrons and nuclei, respectively, while Φ and Θ are the electron wave function and nuclear vibration wave function, respectively, and ${\hat{H}}^{BO}$ is the nonadiabatic coupling. It should be noted that the spin–orbit coupling term ${\hat{H}}^{SO}$ can be neglected due to the extremely small spin–orbit coupling between the first singlet and triple excited states of pure organic fluorophores [31,32]. Consequently, *k*_nr_ between the first excited state (S_1_) and the ground state (S_0_) is defined as

(7) $$k_{\mathrm{nr}}=\frac{2\pi }{\hbar }\sum_{kl}R_{kl}Z_{i}^{-1}\sum_{vu}e^{-\beta E_{iv}}\langle \Theta_{fu}|{\hat{P}}_{fk}|\Theta_{iv}\rangle \langle \Theta_{iv}|{\hat{P}}_{fl}|\Theta_{fu}\rangle \delta (E_{iv}-E_{fu}).$$

Here, *Z_i_* is the partition function, $R_{kl}=\langle \Phi_{f}|{\hat{P}}_{fk}|\Phi_{i}\rangle \langle \Phi_{i}|{\hat{P}}_{fl}|\Phi_{f}\rangle$ denotes the nonadiabatic electronic coupling and ${\hat{P}}_{fk}=-i\hbar \frac{\partial }{\partial Q_{fk}}$ is the normal momentum operator of the *k*th mode in the final electronic state. By Fourier transforming on the delta function, Equation (7) can be converted to

(8) $$k_{\mathrm{nr}}=\sum_{kl}\frac{1}{\hbar^{2}}R_{kl}\int_{-\infty }^{\infty }dte^{i\omega_{\mathrm{if}}t}\left[Z_{i}^{-1}\rho_{\mathrm{nr},kl}\left(t,T\right)\right].$$

Here, $\rho_{\mathrm{ic},kl}(t,T)=\mathrm{Tr}({\hat{P}}_{fk}e^{-i\tau_{f}{\hat{H}}_{f}}{\hat{P}}_{fl}e^{-i\tau_{i}{\hat{H}}_{i}})=\int_{-\infty }^{\infty }dx\langle x|{\hat{P}}_{fk}e^{-i\tau_{f}{\hat{H}}_{f}}{\hat{P}}_{fl}e^{-i\tau_{i}{\hat{H}}_{i}}|x\rangle$ represents the thermal vibration correlation function (TVCF) in the nonadiabatic process [31,33,34,35].

### 3.3. Computational Details

The geometry optimizations of the studied fluorophores in S_0_ were conducted with the density functional theory (DFT) method using the long-range corrected hybrid functional CAM-B3LYP along with the 6-31G(d) basis set, followed by vibrational frequency calculations to verify the stability of the conformations. Previous works have demonstrated the applicability of this approach in accurately determining the optical properties of NIR-II dyes [36,37]. To characterize the photoabsorption performance of the dyes, molecular absorption spectra were determined by calculating the lowest 10 singlet–singlet transitions. Subsequently, the structural optimizations of S_1_ for subsequent frequency calculations were performed. All of the above simulations were carried out with the time-dependent density functional theory (TD-DFT) method at the same theoretical level as the geometry optimization in Gaussian 16 [38]. To consider the influence of the surrounding environments, the integral equation formalism for the polarizable continuum model (IEFPCM) [39,40] was used to describe the experimental aqueous solution. In addition, the dispersion effect was accounted for by incorporating an empirical dispersion correction term in the calculations through utilizing DFT-D3 [41].

Based on the geometric and electronic structure information obtained, the rate constants of the excited state decay, including the radiative and nonradiative decay rates from S_1_ to S_0_, were evaluated. This analysis was conducted using the TVCF method applied in MOMAP [42]. Additionally, to provide a visual representation on the transition properties of the fluorophores, distributions of the holes and electrons as well as the intramolecular electronic transfer analyses were performed with Multiwfn [43], which enabled a comprehensive understanding of the underlying electronic transferring processes [44,45].

## 4. Conclusions

In summary, this study investigated the planarization-to-twisting strategy, which was applied to a series of D-A-D molecular fluorophores by utilizing the TVCF theory coupled with DFT and TD-DFT calculations. By tactfully incorporating the ortho-positioned alkyl units on thiophene into the coplanar monomer BBTD-1, three fluorophores with twisted molecular backbones were theoretically designed. Photophysical characterizations of the studied fluorophores revealed that as the molecular distortion increased, the HOMO–LUMO gap increased, and the optical spectra were blue-shifted. Notably, the investigated dyes exhibited fluorescent peaks exceeding 900 nm, confirming their potential as NIR-II fluorophores. Furthermore, the conformational twisting largely accelerated the fast radiative decay rate while simultaneously suppressing the nonradiative decay rate, resulting in significant improvements in the luminescent efficiency. In comparison with the planar structural BBTD-1, the FQE increased monotonically by at least fivefold in the twisted molecular skeletons. Accordingly, molecular distortion in the BBTD-*n* compounds not only induced a hypsochromic shift on the photophysical spectrum but also facilitated increased luminescent efficiency and thus enhanced the fluorescence brightness. This demonstrates the effectiveness of the twisting strategy in enhancing the photophysical properties of these fluorophores, paving the way for their application in advanced imaging technologies.

Most importantly, the optimal fluorophore, BBTD-4 with a twisted construction, simultaneously possessed NIR-II emission characteristics at 980 nm and a high fluorescence brightness of 16.59 M^−1^cm^−1^. The balanced NIR-II fluorescence behavior and satisfactory brightness exhibited in BBTD-4 can be attributed to the enhanced adiabatic excitation energy and reduced nonadiabatic electronic coupling between the S_1_ and S_0_ states, originating from moderate distortion of the molecular skeleton. Therefore, BBTD-4 is anticipated to be an excellent candidate for NIR-II fluorescence imaging in biomedical applications.

Relative to the previously reported NIR-II chromophores with twisted skeleton by incorporating the EDOT or PDOT moieties, the alkyl-attached spacers is advantageous on the simplified synthesis, cost-effectiveness, and the fine-tuned steric hindrance. This modification provides a versatile approach to manipulate the electronic and optical characteristics of the materials, which opens an efficient shortcut to the rational design of NIR-II chemosensors for future applications. Our work not only presents an structure-property relationships on the studied NIR-II fluorophore, but also highlights an intelligent and rational planar-twisted molecular engineering tactic to the development of highly efficient NIR-II fluorophores for bioimaging utilization.

In the present study, we focused exclusively on the photophysical properties of individual NIR-II fluorophores using an implicit solvent model. However, it is important to note that NIR-II fluorophores are typically affected by the surrounding solvent, solute molecules and even by interactions within aggregated states. Thus, a comprehensive understanding of their photophysical properties requires considering the effects of intermolecular interactions, which are known to play a crucial role in modulating the molecular structure and optical characteristics. This requires further research on the micro-environment-dependent luminescent performance of the chromophore and constructing more precise models in practical applications.

## Figures and Tables

**Table 1:** The excited energy (*E*_Abs_, in eV), absorption wavelength (*λ*_Abs_, in nm), oscillator strength (*δ*_Abs_, in a.u.), transition electric dipole moments (*μ*_01_, in Debye) and the corresponding transition nature of the first molecular excited state, as well as the emission energy (*E*_Emi_, in eV), emission wavelength (*λ*_Emi_, in nm) and the corresponding transition electric dipole moments (*μ*_10_, in Debye) for BBTD-*n* (*n* = 1, 2, 3, 4). H and L represent the HOMO and LUMO, respectively.

Molecule	*E*_Abs_	*λ*_Abs_	*δ*_Abs_	*μ*_01_	Transition Nature	*E_Emi_*	*λ*_Emi_	*μ*_10_
BBTD-1	1.46	848	1.13	14.28	H→L 95%	0.81	1522	18.70
BBTD-2	1.87	662	0.82	10.77	H→L 86%	1.04	1192	15.55
BBTD-3	1.96	634	0.71	9.78	H→L 81%	1.08	1153	14.88
BBTD-4	2.36	524	0.31	5.87	H→L 52%	1.27	980	12.40
					H-2→L 44%			

## Data Availability

The raw data supporting the conclusions of this article will be made available by the authors on request.

## References

[1] Hong G., Antaris A.L., Dai H. (2017). Near-infrared fluorophores for biomedical imaging. Nat. Biomed. Eng..

[2] Stabley D.R., Oh T., Simon S.M., Mattheyses A.L., Salaita K. (2015). Real-time fluorescence imaging with 20 nm axial resolution. Nat. Commun..

[3] Liu X., Yu B., Shen Y., Cong H. (2022). Design of NIR-II high performance organic small molecule fluorescent probes and summary of their biomedical applications. Coord. Chem. Rev..

[4] Wang F., Zhong Y., Bruns O., Liang Y., Dai H. (2024). In vivo NIR-II fluorescence imaging for biology and medicine. Nat. Photonics.

[5] Li D., Deng X., Xu Z., Wang D., Xu G., Zhang P., Qiu P., Xie W., Wang D., Tang B.Z. (2023). Molecular Engineering of NIR-II AIE Luminogen Excited at 1700 nm for Ultradeep Intravital Brain Two-Photon Fluorescence Imaging. Adv. Funct. Mater..

[6] Zhang L.e., Liu C., Zhou S., Wang R., Fan Q., Liu D., Wu W., Jiang X. (2020). Improving Quantum Yield of a NIR-II Dye by Phenylazo Group. Adv. Healthc. Mater..

[7] Hu Z., Fang C., Li B., Zhang Z., Cao C., Cai M., Su S., Sun X., Shi X., Li C. (2020). First-in-human liver-tumour surgery guided by multispectral fluorescence imaging in the visible and near-infrared-I/II windows. Nat. Biomed. Eng..

[8] De Ruysscher D., Niedermann G., Burnet N.G., Siva S., Lee A.W., Hegi-Johnson F. (2019). Radiotherapy toxicity. Nat. Rev. Dis. Primers.

[9] Sorolla M.A., Parisi E., Sorolla A. (2020). Determinants of sensitivity to radiotherapy in endometrial cancer. Cancers.

[10] Sun B., Luo C., Cui W., Sun J., He Z. (2017). Chemotherapy agent-unsaturated fatty acid prodrugs and prodrug-nanoplatforms for cancer chemotherapy. J. Control Release.

[11] Liang P., Huang X., Wang Y., Chen D., Ou C., Zhang Q., Shao J., Huang W., Dong X. (2018). Tumor-microenvironment-responsive nanoconjugate for synergistic antivascular activity and phototherapy. ACS Nano.

[12] Xie Z., Fan T., An J., Choi W., Duo Y., Ge Y., Zhang B., Nie G., Xie N., Zheng T. (2020). Emerging combination strategies with phototherapy in cancer nanomedicine. Chem. Soc. Rev..

[13] Cai Y., Wei Z., Song C., Tang C., Han W., Dong X. (2019). Optical nano-agents in the second near-infrared window for biomedical applications. Chem. Soc. Rev..

[14] Li C., Chen G., Zhang Y., Wu F., Wang Q. (2020). Advanced Fluorescence Imaging Technology in the Near-Infrared-II Window for Biomedical Applications. J. Am. Chem. Soc..

[15] Schmidt E.L., Ou Z., Ximendes E., Cui H., Keck C.H.C., Jaque D., Hong G. (2024). Near-infrared II fluorescence imaging. Nat. Rev. Method Prime..

[16] Antaris A.L., Chen H., Cheng K., Sun Y., Hong G., Qu C., Diao S., Deng Z., Hu X., Zhang B. (2016). A small-molecule dye for NIR-II imaging. Nat. Mater..

[17] Chen H., Liu L., Qian K., Liu H., Wang Z., Gao F., Qu C., Dai W., Lin D., Chen K. (2022). Bioinspired large Stokes shift small molecular dyes for biomedical fluorescence imaging. Sci. Adv..

[18] Lei Z., Zhang F. (2021). Molecular Engineering of NIR-II Fluorophores for Improved Biomedical Detection. Angew. Chem. Int. Ed..

[19] Shao J., Wang G., Wang K., Yang C., Wang M. (2015). Direct arylation polycondensation for efficient synthesis of narrow-bandgap alternating D–A copolymers consisting of naphthalene diimide as an acceptor. Polym. Chem-UK..

[20] Ren T.B., Wang Z.Y., Xiang Z., Lu P., Lai H.H., Yuan L., Zhang X.B., Tan W. (2021). A general strategy for development of activatable NIR-II fluorescent probes for in vivo high-contrast bioimaging. Angew. Chem. Int. Ed..

[21] Zhang X., Wang H., Antaris A.L., Li L., Diao S., Ma R., Nguyen A., Hong G., Ma Z., Wang J. (2016). Traumatic brain injury imaging in the second near-infrared window with a molecular fluorophore. Adv. Mater..

[22] Wan H., Ma H., Zhu S., Wang F., Tian Y., Ma R., Yang Q., Hu Z., Zhu T., Wang W. (2018). Developing a bright NIR-II fluorophore with fast renal excretion and its application in molecular imaging of immune checkpoint PD-L1. Adv. Funct. Mater..

[23] Yang Q., Hu Z., Zhu S., Ma R., Ma H., Ma Z., Wan H., Zhu T., Jiang Z., Liu W. (2018). Donor engineering for NIR-II molecular fluorophores with enhanced fluorescent performance. J. Am. Chem. Soc..

[24] Ma H., Liu C., Hu Z., Yu P., Zhu X., Ma R., Sun Z., Zhang C.-H., Sun H., Zhu S. (2020). Propylenedioxy thiophene donor to achieve NIR-II molecular fluorophores with enhanced brightness. Chem. Mater..

[25] Zhang T., Jiang Y., Niu Y., Wang D., Peng Q., Shuai Z. (2014). Aggregation effects on the optical emission of 1, 1,2,3,4,5-hexaphenylsilole (HPS): A QM/MM study. J. Phys. Chem. A..

[26] Zheng X., Peng Q., Zhu L., Xie Y., Huang X., Shuai Z. (2016). Unraveling the aggregation effect on amorphous phase AIE luminogens: A computational study. Nanoscale.

[27] Peng Q., Shuai Z. (2021). Molecular mechanism of aggregation-induced emission. Aggregate.

[28] Luo Y., Guo Y., Shou X., Chen Z., Xu Z., Tang D. (2022). Investigate the Relationship between Structure and Triplet Potential Energy Surface to Control the Phosphorescence Quantum Yield of Platinum (II) Complex: A Theoretical Investigation. Inorg. Chem..

[29] Peng Q., Fan D., Duan R., Yi Y., Niu Y., Wang D., Shuai Z. (2017). Theoretical study of conversion and decay processes of excited triplet and singlet states in a thermally activated delayed fluorescence molecule. J. Phys. Chem. C..

[30] Xie Y., Zhang T., Li Z., Peng Q., Yi Y., Shuai Z. (2015). Influences of Conjugation Extent on the Aggregation-Induced Emission Quantum Efficiency in Silole Derivatives: A Computational Study. Chem.-Asian J..

[31] Zeng Y., Niu Y., Peng Q., Zheng X. (2022). Origin of Nonmonotonical Variation of Luminescence Efficiency under Pressure in Organic Molecule. J. Phys. Chem. A..

[32] Jiao Y., Dong X., Ran X., Deng Q., Xiao H., Wang Z., Zhang T. (2023). Theoretical characterization of two-photon fluorescent probes for nitric oxide detection: Sensing mechanism, photophysical properties and protonation effects. Phys. Chem. Chem. Phys..

[33] Niu Y., Peng Q., Deng C., Gao X., Shuai Z. (2010). Theory of excited state decays and optical spectra: Application to polyatomic molecules. J. Phys. Chem. A.

[34] Peng Q., Yi Y., Shuai Z., Shao J. (2007). Excited state radiationless decay process with Duschinsky rotation effect: Formalism and implementation. J. Chem. Phys..

[35] Zhang K., Wang X., Zhang Q., Wu Z., Li X., Mu Q., Fan J., Wang C.-K., Lin L. (2022). Insights on isomeric emitters with thermally activated delayed fluorescence: Comparison between solvent and crystal state. Spectrochimica Acta A..

[36] Salzner U., Aydin A. (2011). Improved Prediction of Properties of π-Conjugated Oligomers with Range-Separated Hybrid Density Functionals. J. Chem. Theory Comput..

[37] Zhang L., Zou L.-Y., Guo J.-F., Wang D., Ren A.-M. (2015). A theoretical study of a series of novel two-photon nitric oxide (NO) fluorescent probes based on BODIPY. New J. Chem..

[38] Frisch M.J., Trucks G.W., Schlegel H.B., Scuseria G.E., Robb M.A., Cheeseman J.R., Scalmani G., Barone V., Petersson G.A., Nakatsuji H. (2016). Gaussian 16 Rev. C.01.

[39] Cancès E., Mennucci B., Tomasi J. (1997). A new integral equation formalism for the polarizable continuum model: Theoretical background and applications to isotropic and anisotropic dielectrics. J. Chem. Phys..

[40] Tomasi J., Mennucci B., Cammi R. (2005). Quantum mechanical continuum solvation models. Chem. Rev..

[41] Grimme S., Antony J., Ehrlich S., Krieg H. (2010). A consistent and accurate ab initio parametrization of density functional dispersion correction (DFT-D) for the 94 elements H-Pu. J. Chem. Phys..

[42] Niu Y., Li W., Peng Q., Geng H., Yi Y., Wang L., Nan G., Wang D., Shuai Z. (2018). MOlecular MAterials Property Prediction Package (MOMAP) 1.0: A software package for predicting the luminescent properties and mobility of organic functional materials. Mol. Phys..

[43] Lu T., Chen F. (2012). Multiwfn: A multifunctional wavefunction analyzer. J. Comput. Chem..

[44] Zhang M., Fu H., Hu W., Leng J., Zhang Y. (2022). Versatile Dicyanomethylene-Based Fluorescent Probes for the Detection of β-Amyloid in Alzheimer’s Disease: A Theoretical Perspective. Int. J. Mol. Sci..

[45] Liu Z., Lu T., Chen Q. (2020). An sp-hybridized all-carboatomic ring, cyclo [18] carbon: Electronic structure, electronic spectrum and optical nonlinearity. Carbon.

